# The Effect of Anxiety and Nutritional Habits on the Body Distortion of Athletes

**DOI:** 10.3390/nu17040682

**Published:** 2025-02-14

**Authors:** Maria Isabel Ramírez-Goerke, José Francisco Tornero-Aguilera, Alexandra Martín-Rodríguez, Vicente Javier Clemente-Suárez

**Affiliations:** 1 Department of Pharmacy and Nutrition, Faculty of Health Sciences, Universidad Europea de Madrid, Villaviciosa de Odón, 28670 Madrid, Spain; mariaisabel.ramirez@universidadeuropea.es; 2 KOS Generating Health, 45007 Toledo, Spain; 3 Faculty of Medicine, Health and Sports, Universidad Europea de Madrid, Villaviciosa de Odón, 28670 Madrid, Spain; sandra.martin.rodriguez8@gmail.com (A.M.-R.); vctxente@yahoo.es (V.J.C.-S.); 4 Faculty of Applied Social Sciences and Communications, International Business University (UNIE), 28015 Madrid, Spain; 5 Grupo de Investigación en Cultura, Educación y Sociedad, Universidad de la Costa, Barranquilla 080002, Colombia;

**Keywords:** anxiety, athletes, nutritional habits, body composition, body distorsion

## Abstract

**Background:** Anxiety disorders have been rising globally, particularly among adolescents and women. However, the relationship between diet, psychological traits, and anxiety levels in athletes remains underexplored. **Objectives:** This study aimed to analyze the nutritional and psychological differences between athletes with varying anxiety levels, hypothesizing that higher anxiety correlates with unhealthier dietary habits, greater body distortion, and less adaptive psychological profiles. **Methods:** A total of 58 athletes (23 women, 35 men), aged 18 to 45 years (mean age = 30.2 years), participated in this cross-sectional study. Data were collected using validated online questionnaires, including the Big Five Inventory, Spielberger State–Trait Anxiety Inventory (STAI), Acceptance and Action Questionnaire-II (AAQ-II), UCLA Loneliness Scale, and Eating Disorder Inventory (EDI), as well as surveys assessing nutritional habits and physical activity levels. Statistical analyses were conducted using SPSS (v24.0), with independent *t*-tests to compare differences between higher and lower anxiety groups (*p* < 0.05). **Results:** It has beenindicated that higher anxiety was associated with greater neuroticism, lower psychological flexibility, and higher eating disorder symptomatology, while better sleep quality and psychological profiles correlated with lower anxiety levels. Additionally, athletes who cooked their own meals exhibited higher anxiety, whereas greater water intake and whole grain consumption were linked to lower anxiety. More frequent and intense training, particularly weight training, was also associated with reduced anxiety. **Conclusion:** This study concludes that anxiety in athletes is influenced by multiple lifestyle factors, including sleep quality, dietary habits, psychological traits, and exercise patterns. These findings emphasize the need for holistic approaches integrating nutrition, psychological interventions, and structured physical training to manage anxiety in athletes.

## 1. Introduction

Anxiety is a global disorder whose incidence has been constantly increasing over the years. From the 1990’s to 2019, anxiety cases increased from 31.13 million to 45.82 million, which represents a 47.19% increase. In only one year, from 2018 to 2019, there was an increase of 0.6 million cases. The statistics show that people between ten and fourteen years old have the highest incidence of anxiety and are followed by people between thirty-five and forty-four years old. In terms of gender, women are more likely to present with an anxiety disorder than men [1]. This is reflected in both higher rates of diagnosed anxiety disorders and self-reported anxiety scores, with women typically reporting two to three times greater levels of anxiety than men [2,3,4,5].

Anxiety is a multifactorial disorder, meaning that it can be influenced by many factors, like food. There is evidence that indicates a relationship between decreased anxiety symptoms and healthy eating habits. A diet intervention is a treatment that does not involve a high risk and it is cost-effective [6]. Sadly, this is often not considered when treating anxiety patients [7]. On the other hand, the lack of vitamins or minerals might be related to the development of diseases like sleep disturbance, mood disorders, and anxiety disorders among others [8], showing that anxiety and diet are closely related, with diet being a helpful tool in treating this disorder, but also being a factor (inadequate diet) that causes such a disorder [9]. On the other hand, inflammation could also play a role in anxiety, since pro-inflammatory cytokines have also been associated with emotional regulation [10].

As stated before, diet is key to mental health, and sometimes athletes have trouble accepting their bodies, which can generate eating disorders, potentially leading to anxiety [11]. However, the relationship between sports and body distortion (i.e., a misperception of one’s body shape or size, often leading to dissatisfaction and maladaptive behaviors) may vary depending on factors such as competition level, training background, sport type, age, and gender. Adolescents are usually more prone to developing these disorders [12], showing a distinctly altered perception of their bodies [13]. Men who train intensely tend to have a different body image than men who do not train [11]. Athletes involved in aesthetic sports (i.e., sports emphasizing physical appearance, body aesthetics, and artistic presentation, such as gymnastics, figure skating, diving, bodybuilding, and ballet) are more likely to develop body image dissatisfaction than athletes participating in non-aesthetic sports [14]. Athletes are usually subjected to a lot of pressure, which could trigger anxiety. Individuals scoring high on anxiety sensitivity exhibit an increased prevalence of eating disorder symptoms when assessed using the Eating Disorder Inventory (EDI) [15].

The relationship between body distortion and anxiety among athletes has been suggested in previous research but remains underexplored. Although body dissatisfaction has been linked to anxiety in the general population, studies focusing on athletes—particularly in relation to their psychological and nutritional profiles—are scarce. This study aimed to analyze the nutritional and psychological differences in athletes with varying anxiety levels and to assess whether body distortion is significantly associated with anxiety in this population.

The initial hypothesis was that athletes with higher anxiety levels would present unhealthier nutritional habits, a less adaptive psychological profile, and a higher level of body distortion compared to athletes with lower anxiety levels. This study provides further insight into the mechanisms by which psychological flexibility, nutritional patterns, and training behaviors influence anxiety and body perception in athletes, contributing to the development of more effective interventions in sports psychology and nutrition.

## 2. Materials and Methods

A total of 58 athletes (23 women and 35 men) with a mean age of 30.2 years (mean height of 167.2 ± 9.7 cm and mean weight of 63.8 ± 13.1 kg) were analyzed in this study. Participants were exclusively athletes engaged in aesthetic sports, including gymnastics, figure skating, diving, bodybuilding, and ballet. The sample size was determined using G*Power software, with a statistical power of 0.80 and an alpha level of 0.05, based on prior studies analyzing anxiety, body image, and nutritional habits in athletes. Recruitment was conducted through sports academies, athletic clubs, and online athlete networks.

Inclusion Criteria:

- Athletes actively training and competing in aesthetic sports (gymnastics, figure skating, diving, bodybuilding, ballet).
- Aged between 18 and 40 years to ensure a homogeneous adult sample.
- Minimum training frequency of four sessions per week for at least one year to ensure participants had structured training routines.

Exclusion Criteria:

- History of clinically diagnosed eating disorders, severe anxiety disorders, or major psychiatric conditions to prevent confounding psychological effects.
- Use of pharmacological or dietary treatments specifically aimed at anxiety management.
- Participation in non-aesthetic sports or irregular training schedules.

The study received approval from the University Ethics Committee (CIPI/21/082), adhering to the Helsinki Declaration (revised in Brazil, 2013) for ethical research involving human subjects.

### 2.1. Procedures

The participants were interviewed via an online questionnaire following previous methodologies [16]. The following variables were analyzed.

*Psychological profile:* This study used a shorter version of the Spanish Big Five Inventory (alpha coefficient 0.73) [17] which included 44 items of personality traits. This version encompassed questions about five key aspects of personality: neuroticism, openness to experience, agreeableness, extraversion, and conscientiousness. It incorporates 10 items which are replied with a 5-point Likert scale, where 5 means completely agree and 1 means completely disagree. A condensed version of the Spanish Spielberger State–Trait Anxiety Inventory (alpha coefficient 0.93) [18] was used. This included 6 items measuring anxiety with responses on a 4-point Likert scale where 4 means very much and 1 means not at all. The Acceptance and Action Questionnaire II (alpha coefficient.84 (0.78–0.88)) [19] in Spanish was applied. With this test, psychological inflexibility or experiential avoidance is studied using 7 items, with replies given via a 7-point Likert scale, where 7 means always and 0 means never. To measure loneliness, a short version of the UCLA Loneliness Scale (alpha coefficient 0.94) [20] in Spanish was used. This consisted of 3 items, with replies given via a 3-point Likert scale, where 3 means frequently and 1 means never. EDI was also applied, which consisted of 91 items, with replies given via a 6-point Likert scale. It consisted of 11 subscales, and was designed to evaluate behavioral and psychological characteristics usually seen in bulimia and anorexia nervosa [21,22,23].

*Physical Activity Habits:* The questionnaire included the following questions.

During this past year, how many times have you been injured? How many training sessions do you do per week? What is your best score in the 2000 m? What is your mean training time in minutes? From your weekly training time, how many minutes are spent in zone 1? From your weekly training time, how many minutes are spent in zone 2? From your weekly training time, how many minutes are spent in zone 3? How many minutes a week are dedicated to training with self-load? How many minutes a week are dedicated to training with weight? How many minutes a week are dedicated to training strength with weights?

*Nutritional Habits:* A previously used questionnaire, where the quantity and consumption frequency of each food was asked (food consumption frequency questionnaire) [16], was evaluated.

### 2.2. Statistical Analysis

Statistical analyses were conducted using IBM SPSS Statistics 24.0 (SPSS Inc., Chicago, IL, USA). Descriptive statistics (mean and standard deviation) were computed for all study variables to summarize demographic, psychological, and nutritional data.

To assess the normality and homogeneity of variance, the Kolmogorov–Smirnov test was applied to each variable, ensuring the appropriateness of subsequent parametric tests. Given that the data distribution met the assumptions for parametric analysis, an independent samples t-test was used to compare differences between the higher and lower anxiety groups in nutritional habits, psychological traits, body image perception, and physical activity levels. The *t*-test results included t-values, *p*-values, and 95% confidence intervals to determine statistical significance. A significance level of *p* < 0.05 was set in accordance with standard statistical conventions.

This analytical approach allowed for a robust comparison of anxiety-related differences across multiple lifestyle, dietary, and psychological variables, providing valuable insights into their potential influence on anxiety levels among athletes.

## 3. Results

Upon reviewing the obtained data, the analysis revealed some significant differences in the backgrounds of the participants. Table 1 shows that the lower anxiety group presented significantly higher values in the sleep quality variable than the lower anxiety group.

Regarding the psychological profile it is noted that individuals with a neuroticism physiological profile have higher anxiety. Individuals from the higher anxiety group showed significantly higher results on the AAQII questionnaire and on the STAI (Table 2).

Results showed that individuals who cook most of their meals have higher anxiety than those who do not, and individuals who drink more water a day also have less anxiety (Table 3). Individuals with a higher egg intake have more anxiety, and individuals with a higher whole-grain cereal intake have less anxiety. Individuals with higher EDI scores showed significantly higher anxiety than individuals with lower EDI scores. Individuals who identified themselves with higher silhouette numbers also have significantly higher anxiety levels than those who identified with lower silhouette numbers (Table 4). People who train more time per week have less anxiety than those who train less time per week. Additionally, individuals who train more time with weight also have less anxiety than those who train less time with weights (Table 5).

In summary, these results underscore the multifaceted nature of anxiety and its interaction with lifestyle factors, highlighting the importance of holistic approaches in managing and mitigating anxiety-related symptoms.

## 4. Discussion

The aim of this study was to analyze the nutritional and psychological differences in athletes with different anxiety levels. The initial hypothesis was accepted since athletes with higher anxiety levels presented higher unhealthier nutritional habits, a less adaptive psychological profile, and a higher body distortion than athletes with lower anxiety levels.

In the present study, the lower anxiety group presented significantly higher values in the sleep quality and quantity variables than the lower anxiety group. A decreased amount of sleep hours has been associated with anxiety [24]. Many studies have found a reciprocal relationship between anxiety and sleep alteration since one could be the result or the cause of the other, and because they are interwoven in time [25,26,27]. Insomnia and anxiety have been shown to have a bidirectional relationship [28]. It was found that a reduced sleep duration can predict anxiety disorders [24]. Our results agree with former studies, showing that participants with a better sleep quantity and quality present with less anxiety.

Regarding the psychological profile, individuals with neuroticism present higher anxiety. The association between personality and emotional disorders is complicated [29]. Individuals with neuroticism tend to be more prone to developing anxiety [30,31,32]. Other research has concluded that neuroticism is strongly related to anxiety in the general population [29], which agrees with our results. Neuroticism alludes to subjects that are presented as anxious or irritable, and that is why they demonstrate augmented activation in the neural structures related to sensitivity to threat and punishment [33]. Neurotic individuals are more emotionally sensitive to stimuli, and it takes longer for them to go back to the pre-stimulated state [34]. Individuals with this personality trait tend to stress easier, preoccupy excessively, and react in a negative way that overpasses the gravity of the situation, making them more prone to develop anxiety [35]. This information and the results of the previously mentioned studies agree with and explain our results.

Regarding the AAQII questionnaire and the STAI, individuals from the higher anxiety group showed significantly higher results in both. Individuals with lower psychological flexibility have a higher risk of anxiety [36]. Experiential avoidance seeks to control the way or frequency of aversive private experiences [37] and has been associated with anxiety [38,39,40,41,42,43,44,45,46,47,48,49,50,51,52,53,54]. This explains why the participants with higher results on the AAQII questionnaire belonged to the higher anxiety group. Since the STAI questionnaire measures anxiety, it corroborates the fact that participants with higher STAI scores belong to the group of individuals with higher anxiety.

In the present study, individuals with higher EDI scores showed significantly higher anxiety levels. Research [53,54,55,56,57] has shown that anxiety could be a predecessor of image distortion. The literature states that both disorders might have common ethological factors, which can make a person more prone to either disorder [58]. This agrees with other studies like Behar et al. [59], where individuals with eating disorders showed 40.7% higher anxiety levels compared to individuals who were not suffering from them. Additionally, Merida et al. [60] showed that 54% of patients with eating disorders presented with anxiety.

Furthermore, individuals who identified themselves with a higher silhouette number also showed significantly higher anxiety levels. A systematic review and meta-analysis also showed a correlation between anxiety and body dissatisfaction in men [61]. Another research study showed a relationship between anxiety and drive for thinness [62]. Our results also agree with this and other studies [61,63], which showed that adolescent girls with body dissatisfaction presented higher anxiety levels.

Regarding nutritional items analyzed, in the cooking their own meals item, participants who cook most of their meals have higher anxiety than the ones who do not. There is no literature available on this matter, but it could be inferred that the fact of deciding what to cook every day, plus it being time-consuming, could increase the anxiety of the subjects, in contrast to individuals who just order in, or have someone who cooks for them.

According to water intake, it was found that individuals who drink more water a day have less anxiety. Daily habits can have a big influence on anxiety [43]. When the water intake increases, the result is a dilution of plasma noradrenaline. Activation of the norepinephrine-vasopressinergic system and subsequently HPA(hypothalamus–pituitary axis) is induced by psychosomatic depression. It is believed that depressive disorders are caused by an increase in the vasopressinergic activation of HPA [44]. There is only one study that evaluates water intake and anxiety [43], and their results agree with ours.

Regarding egg intake, subjects with a higher egg intake present with more anxiety. However, the literature states that there is no association between egg intake and psychological disorders [43]. Additionally, our study shows that individuals with a higher whole-grain cereal intake have less anxiety. Cognitive decline encompasses mental health conditions like anxiety [45,46]. Insulin resistance and inflammation can cause anxiety [47], and whole grains decrease inflammatory biomarkers [48] and influence the postprandial glucose metabolism [49,50,51], which could affect insulin sensitivity. This, in turn, could regulate better glucose-related mood changes [48]. A systematic review showed that there was an association between reduced anxiety and a higher whole-grain intake [52], which agrees with our results.

Finally, our results showed that athletes who engage in longer training sessions per week experience lower anxiety levels compared to those who train less frequently. Additionally, athletes who dedicate more time to weight training also exhibit significantly lower anxiety levels than those who engage in less resistance training. This aligns with previous research indicating that physical exercise triggers the release of endorphins and endogenous opioids, which contribute to feelings of euphoria, stress reduction, and overall psychological well-being [64]. Furthermore, regular exercise has been shown to enhance cognitive function, which plays a crucial role in regulating mood and reducing anxiety symptoms [65]. The positive impact of sports participation on anxiety has been widely supported by the existing literature [66], reinforcing the importance of structured and consistent physical activity—particularly weight training—as a valuable tool in anxiety management among athletes in aesthetic sports.

### 4.1. Limitations of the Study

The main limitation of this study is the small sample size of 23 women and 35 men, which may not be representative of the broader population of athletes. Another limitation is the cross-sectional design of the study, which limits the ability to establish causal relationships between variables. Future research could benefit from increasing the sample size to improve generalizability, and using a longitudinal design to explore the causal effects of anxiety on nutritional habits, psychological profiles, and body distortion in athletes. Future studies might also focus on specific athlete subgroups, such as elite athletes or adolescent athletes, to provide more targeted insights

### 4.2. Practical Applications

The findings of this study suggest several practical strategies for reducing anxiety among athletes by addressing key lifestyle factors, including sleep quality, nutrition, psychological flexibility, and physical activity.

1. Optimizing Sleep Quality and Quantity

Adequate sleep plays a crucial role in anxiety regulation. Our results showed that athletes with better sleep quality and longer sleep duration exhibited lower anxiety levels. Given the bidirectional relationship between anxiety and sleep disturbances, implementing structured sleep hygiene practices, ensuring consistent sleep schedules, and optimizing pre-sleep routines can contribute to better mental well-being and athletic performance.

2. Promoting Nutritional Interventions

Healthy dietary habits are strongly linked to anxiety regulation. Our study found that higher water and whole grain intake was associated with lower anxiety levels, supporting previous research that suggests nutritional choices can modulate stress responses. Whole grains help reduce inflammation and stabilize glucose metabolism, which may improve mood and cognitive function. Encouraging athletes to maintain a balanced diet rich in essential nutrients—with adequate hydration and fiber intake—can be an effective, low-risk, and cost-effective strategy for anxiety management. Conversely, our study revealed that athletes who cooked their own meals reported higher anxiety levels. While the literature on this specific relationship is scarce, it is possible that the added stress of meal planning and preparation contributes to anxiety. Future interventions could explore strategies to simplify meal preparation, offer guided meal planning, or provide nutritional coaching to mitigate this stressor.

3. Enhancing Psychological Flexibility and Reducing Body Image Distortions

Athletes with higher neuroticism scores, lower psychological flexibility, and greater body dissatisfaction were more prone to anxiety. Psychological interventions—such as Acceptance and Commitment Therapy (ACT)—can help athletes develop emotional resilience, reduce experiential avoidance, and improve adaptability to stressors. Additionally, educating athletes on body image, promoting a performance-oriented mindset over aesthetic concerns, and integrating sports psychology into training programs can help minimize anxiety linked to body dissatisfaction and disordered eating behaviors.

4. Encouraging Regular Physical Activity and Strength Training

Our results confirmed that athletes who engaged in more frequent and intense training had lower anxiety levels. This aligns with well-established research demonstrating that exercise induces endorphin release, enhances cognitive function, and regulates the autonomic nervous system. Incorporating strength training and aerobic exercise into an athlete’s routine can serve as a natural anxiety-reducing intervention, improving both psychological well-being and athletic performance.

## 5. Conclusions

This study highlights the complex interplay between nutrition, psychological traits, body image, and physical activity in shaping anxiety levels among athletes. Unhealthier dietary habits, lower psychological flexibility, and greater body distortion contribute to higher anxiety. Improved sleep quality and duration were associated with lower anxiety, while individuals with a neuroticism profile and disordered eating behaviors exhibited higher anxiety levels.

Notably, athletes who cooked their own meals reported higher anxiety, suggesting a potential stress factor related to meal planning. In contrast, greater water intake was linked to reduced anxiety levels, supporting research on hydration’s role in stress regulation. Additionally, those who engaged in frequent and intense physical training, particularly weight training, demonstrated lower anxiety levels, reinforcing the mental health benefits of structured exercise. These findings underscore the importance of holistic approaches integrating nutrition, psychological interventions, and exercise to manage anxiety in athletes. Future research should explore causal relationships and targeted interventions to enhance mental resilience and athletic performance.

## Figures and Tables

**Table 1:** Demographic, education, employment, sleep, and social media data.

Variable	Higher Anxiety Group	Lower Anxiety Group	T	*p*	95% Confidence Interval of the Difference	
	Mean ± Standard Deviation	Mean ± Standard Deviation			Inferior	Superior
Age (years)	33 ± 12.1	35 ± 13.2	−0.754	0.453	−7.26689	3.26882
Height (cm)	166.1 ± 8.1	171.5 ± 9.8	−2.838	0.006	−9.08731	−1.60352
Weight (kg)	63.8 ± 12.3	69.8 ± 13.4	−2.192	0.031	−11.42862	−0.55823
BMI	21.9 ± 5.7	23.7 ± 4	−1.773	0.080	−3.86846	0.21977
Sex (M1/F2)	1.4 ± 0.5	1.2 ± 0.4	3.185	0.002	0.10995	0.47459
Education Level (1 elementary school, 2 middle school, 3 high school, 4 professional training, 5 bachelor’s, 6 master’s, 7 PHD)	4.4 ± 1.3	4.8 ± 1.4	−1.413	0.161	−0.97545	0.16482
Job Situation (1 no job, 2 part-time job, 3 full-time job, 4 retired)	2.3 ± 1	2.6 ± 0.9	−1.585	0.117	−0.70125	0.07903
Job Type (1 no job, 2 private job, 3 self-employed, 4 retired)	2.2 ± 0.9	2.2 ± 0.7	0.380	0.705	−0.28183	0.41516
Mean sleep hours a day 5 = 5 or less/hours 10 = 10 h	6.7 ± 1.1	7.2 ± 1.1	−1.973	0.052	−0.92976	0.00319
Sleep quality last night (0 bad sleep quality, 10 maximum sleep quality)	7.1 ± 1.6	8 ± 1.3	−2.757	0.007	−1.45469	−0.23613
Mean steps per day, last week	17,777 ± 50,110.3	10,607 ± 6997.9	0.929	0.356	−8204.26712	22,544.21156
How many hours per day are spent on social media? (30 min per day = 0.5, an hour= 1, hour and 15 min= 1.25...)	5 ± 18.3	2.1 ± 1.2	1.064	0.290	−2.52855	8.35078
Preferred social media (1 WhatsApp, 2 Instagram, 3 Facebook, 4 TikTok, 5 Twitter)	2.3 ± 0.9	2.6 ± 1.1	−1.352	0.180	−0.71363	0.13585

BMI (Body Mass Index), PHD (Doctor of Philosophy).

**Table 2:** Psychological Profile.

Variable	Higher Anxiety Group	Lower Anxiety Group	T	*p* Value	95% Confidence Interval of the Difference	
	Mean ± Standard Deviation	Mean ± Standard Deviation			Inferior	Superior
Extraversion	4 ± 0.8	4.1 ± 0.8	−0.752	0.454	−0.47705	0.21521
Agreeableness	6.5 ± 1.2	7 ± 1.2	−2.020	0.046	−1.01193	−0.00836
Conscientiousness	7 ± 1.3	7.5 ± 1.9	−1.294	0.199	−1.09973	0.23210
Neuroticism	6 ± 1.9	4.7 ± 1.8	3.559	0.001	0.59524	2.10042
Openness to experience	7.4 ± 1.5	7.7 ± 1.6	−0.686	0.494	−0.89590	0.43600
AAQII Psychological inflexibility	23.1 ± 10.1	18.3 ± 7.7	2.551	0.012	1.06253	8.55003
UCLA Loneliness Scale	4.5 ± 1.5	4.2 ± 1.3	1.035	0.303	−0.27058	0.85899
STAI summarized	13.9 ± 2.6	9.1 ± 1.6	10.492	0.000	3.89251	5.71136

AAQII (Acceptance and Action Questionnaire Psychological inflexibility II), UCLA Loneliness Scale (University of California at Los Angeles). STAI summarized (State–Trait Anxiety Inventory).

**Table 3:** Nutritional Data.

Variable	Higher Anxiety Group	Lower Anxiety Group	T	*p* Value	95% Confidence Interval of the Difference	
	Mean ± Standard Deviation	Mean ± Standard Deviation			Inferior	Superior
How many days per week do you eat outside?	1.7 ± 1.3	1.8 ± 1.3	−0.182	0.856	−0.59351	0.49400
How many days do you eat takeout?	1 ± 0.9	1.1 ± 1.2	−0.479	0.633	−0.56003	0.34264
Do you cook most of your meals a day?	2.9 ± 1.3	2.4 ± 1.3	1.987	0.050	0.00004	1.08305
How satisfied are you with your weight?	1.6 ± 0.9	1.9 ± 0.9	−1.284	0.203	−0.62282	0.13393
How many cups of water do you drink per day?	2.8 ± 1.3	3.6 ± 1.3	−2.933	0.004	−1.31921	−0.25373
IF per week of fruit juices and nectars (250 mL)	2.4 ± 1.1	2.4 ± 1	0.141	0.889	−0.40610	0.46794
IF per week of alcoholic beverages (50 mL)	1.3 ± 0.5	1.1 ± 0.3	2.270	0.026	0.02522	0.37961
IF per week of beer (250 mL)	1.5 ± 0.6	1.5 ± 0.6	0.086	0.931	−0.23414	0.25540
IF per week of wine (50 cc)	1.3 ± 0.5	1.3 ± 0.5	0.261	0.795	−0.19224	0.25038
IF per week of soft drinks (250 mL)	1.8 ± 0.7	1.8 ± 0.6	−0.345	0.731	−0.33138	0.23340
IF per week of energy drinks (250 mL)	1.4 ± 0.8	1.4 ± 0.8	−0.324	0.747	−0.38228	0.27521
IF per week of milk (250 mL)	2.5 ± 1.4	2.5 ± 0.9	−0.049	0.961	−0.49894	0.47470
IF per week of fermented dairy (250 mL)	2.4 ± 1.1	2.3 ± 0.9	0.349	0.728	−0.35353	0.50404
IF per week of sweet bakery (1 portion)	2.6 ± 1.3	2.2 ± 0.9	1.766	0.081	−0.05069	0.86036
IF per week of cheese (50 gr)	2.8 ± 1.3	2.7 ± 0.8	0.468	0.641	−0.33545	0.54221
IF per week of eggs (1 egg)	3.6 ± 1.3	3 ± 0.7	2.863	0.005	0.19714	1.09175
IF per week of meat (150 gr)	2.9 ± 1.3	3 ± 1	−0.576	0.566	−0.63131	0.34776
IF per week of fish (150 gr)	2.4 ± 1.1	2.3 ± 0.7	0.729	0.468	−0.24196	0.52216
IF per week of sausage and lunch meat (50 gr)	2.3 ± 1.3	2.2 ± 0.7	0.513	0.609	−0.31891	0.54114
IF per week of legumes (200 gr)	3.1 ± 1.3	3.1 ± 1	−0.168	0.867	−0.54019	0.45614
IF per week of rice (150 gr)	3.1 ± 1.5	3.1 ± 1.1	0.091	0.928	−0.51560	0.56487
IF per week of pasta (150 gr)	2.6 ± 1.2	2.5 ± 0.7	0.492	0.624	−0.30359	0.50359
IF per week of fruit (1 fruit)	3.3 ± 1.3	3.3 ± 1.1	0.254	0.800	−0.45131	0.58377
IF per week of raw vegetables (200 gr)	3.3 ± 1.3	3.2 ± 1.3	0.500	0.618	−0.40898	0.68434
IF per week of cooked vegetables (200 gr)	3.3 ± 1.2	2.8 ± 1.1	1.821	0.072	−0.04098	0.93694
IF per week of bread (1 bun 50 gr)	2.8 ± 1.3	2.9 ± 1.1	−0.269	0.788	−0.57843	0.44027
IF per week of whole grain cereal	2.6 ± 1	3 ± 1.1	−2.174	0.032	−0.89132	−0.04008
IF per week of fast food	1.8 ± 0.8	1.8 ± 0.5	0.000	1.000	−0.27466	0.27466
IF per week of protein shake (300 mL)	2 ± 1.3	2.2 ± 1.2	−0.845	0.400	−0.72686	0.29304
IF per week of gels (40 gr)	1.6 ± 0.8	1.6 ± 0.9	−0.179	0.858	−0.37986	0.31706
IF per week of muesli bars (150 gr)	1.4 ± 0.6	1.6 ± 1.1	−1.374	0.173	−0.61189	0.11163

IF (Intake Frequency).

**Table 4:** Body Satisfaction Data.

Variable	Higher Anxiety Group	Lower Anxiety Group	T	*p* Value	95% Confidence Interval of the Difference	
	Mean ± Standard Deviation	Mean ± Standard Deviation			Inferior	Superior
EDI Results	4.6 ± 4.2	2.1 ± 2.3	3.571	0.001	1.11486	3.91123
Silhouette number you better identify with	17.1 ± 8.9	12.3 ± 9.4	2.460	0.016	0.91153	8.57736
Silhouette number you better identify with	16 ± 9	12.1 ± 10.2	1.890	0.062	−0.19884	7.93217

EDI Results (Eating Disorder Inventory).

**Table 5:** Health and Physical Activity Data.

Variable	Higher Anxiety Group	Lower Anxiety Group	T	*p* Value	95% Confidence Interval of the Difference	
	Mean ± Standard Deviation	Mean ± Standard Deviation			Inferior	Superior
During this past year, how many times have you been injured?	12.6 ± 34	12.7 ± 43.9	−0.014	0.989	−16.51142	16.27760
How many training sessions do you do per week?	5.2 ± 2.7	5.5 ± 2.5	−0.516	0.607	−1.35275	0.79526
Which is your best 200 m mark?	8 ± 1.4	6.8 ± 2.5	1.772	0.085	−0.16836	2.48029
Mean training time in minutes	458.4 ± 325	750.8 ± 496.7	−2.679	0.010	−511.09743	−73.62757
From your weekly training time, how many minutes are spent in zone 1?	152.7 ± 180.8	144.8 ± 112.1	0.180	0.858	−80.64222	96.45391
From your weekly training time, how many minutes are spent in zone 2?	129.1 ± 147.7	181.2 ± 171.8	−1.162	0.251	−142.08967	37.96099
From your weekly training time, how many minutes are spent in zone 3?	88.5 ± 100.4	146.6 ± 120.7	−1.857	0.069	−120.94668	4.80902
How many minutes a week are dedicated to training with self-load?	50.2 ± 67.8	71.5 ± 69.6	−1.097	0.278	−60.20308	17.68584
How many minutes a week are dedicated to training with weights?	35.2 ± 53.3	79.9 ± 100.9	−2.106	0.040	−87.24031	−2.11173
How many minutes a week are dedicated to training strength with weights?	48.3 ± 32.7	51.2 ± 30.5	−0.254	0.801	−25.66765	19.98137

## Data Availability

Data are contained within the article.
